# Internet-Based Cognitive-Behavioral Therapy for Depression: A Feasibility Study for Homecare Older Adults

**DOI:** 10.1093/geroni/igaa057.1194

**Published:** 2020-12-16

**Authors:** Xiaoling Xiang, Yihang Sun, Shawna Smith, Patrick Ho Lam Lai, Joseph Himle

**Affiliations:** 1 University of Michigan, Ann Arbor, Michigan, United States; 2 University of Michigan-Ann Arbor, Ypsilanti, Michigan, United States

## Abstract

This pilot study examined the feasibility of delivering internet-based cognitive behavioral therapy (iCBT) to homebound older adults with symptoms of depression who are recipients of non-medical home care. A feasibility open trial was conducted in the homes of homecare older adults (n=26). When possible, home care workers (HCWs) of older adults (n=13) were recruited to provide external support for iCBT usage. In cases where consistent assistance from the same HCW was not feasible, participants were given the choice of working on the program on their own (n=7) or receiving assistance from a research assistant (RA) (n=6). The mean therapy sessions completed was 4.7 out of 8 total sessions. The mean satisfaction rating was 7.7 (SD=2.9) and 86% would recommend the program to others with depressed mood. Significant reductions in depressive symptoms and anxiety symptoms and improvement on a quality of life measure were observed at post-test. The RA-supported group tended to have the best adherence, satisfaction, and reduction in depressive symptoms, followed closely by the HCW-supported group. The self-guided group had the lowest adherence, satisfaction, and symptom reduction. iCBT is a feasible and acceptable treatment modality for homebound older adults with depressive symptoms and potentially effective. Data from the participant exit interviews suggest a need for refining the existing treatment platform to better meet the needs and capabilities of homebound older adults. Future studies are warranted to examine treatment effectiveness as a function of HCW support.

